# Calycosin alleviates ferroptosis and attenuates doxorubicin-induced myocardial injury via the Nrf2/SLC7A11/GPX4 signaling pathway

**DOI:** 10.3389/fphar.2024.1497733

**Published:** 2024-11-12

**Authors:** Quancheng Han, Jingle Shi, Yiding Yu, Huajing Yuan, Yonghong Guo, Xiujuan Liu, Yitao Xue, Yan Li

**Affiliations:** ^1^ First Clinical Medical College, Shandong University of Traditional Chinese Medicine, Jinan, China; ^2^ Department of Cardiology, Affiliated Hospital of Shandong University of Traditional Chinese Medicine, Jinan, China

**Keywords:** Calycosin, heart failure, ferroptosis, Nrf2/SLC7A11/GPX4 signaling pathway, doxorubicin

## Abstract

**Background:**

Heart failure is primarily characterized by damage to the structure and function of the heart. Ferroptosis represents a form of programmed cell death, and studies indicate that it constitutes one of the primary mechanisms underlying cardiomyocyte death in heart failure. Calycosin, a natural compound derived from astragalus, exhibits various pharmacological properties, including anti-ferroptosis, antioxidant effects, and cardiovascular protection. Nonetheless, the specific role of Calycosin in the treatment of ferroptosis in heart failure remains poorly understood.

**Objective:**

This study aims to elucidate the regulatory effect of Calycosin on ferroptosis and its influence on the treatment mechanisms of heart failure through *in vivo* and *in vitro* experiments.

**Methods:**

A rat model of heart failure was induced using doxorubicin, and the cardiac function was evaluated through cardiac ultrasound examination and NT-Pro BNP detection. Myocardial injury was assessed using H&E staining and Masson staining. The extent of mitochondrial damage was evaluated through transmission electron microscopy. Concurrently, the level of ferroptosis was analyzed by measuring ferroptosis markers, including MDA, ferrous ions, the GSH/GSSG ratio, and GPX4 activity. Subsequently, the molecular mechanism by which Calycosin exerts its therapeutic effects in heart failure was investigated through immunofluorescence and Western blotting. Finally, H9c2 cardiomyocytes were treated with doxorubicin to simulate myocardial injury, and the mechanism by which Calycosin mediates its effects in the treatment of heart failure was further verified through Nrf2 gene silencing.

**Results:**

Calycosin significantly improves cardiac function in rats, reduces serum NT-Pro BNP levels, and alleviates myocardial cell damage. Additionally, it significantly decreases the levels of ferroptosis in myocardial tissue, as confirmed through transmission electron microscopy and the assessment of ferroptosis markers, including MDA, ferrous ions, GSH, and GPX4 activity. At the molecular level, Calycosin exerts its effects by activating the Nrf2/SLC7A11/GPX4 signaling pathway, evidenced by the upregulation of Nrf2, SLC7A11, GPX4, GSS, and GCL protein expression. This process substantially enhances the antioxidant capacity of rat myocardial tissue and effectively suppresses ferroptosis in myocardial cells. The results obtained from both *in vivo* and *in vitro* experiments are consistent. Notably, when Nrf2 is silenced, the protective effect of Calycosin on the myocardium is markedly diminished.

**Conclusion:**

Calycosin effectively treats doxorubicin-induced cardiac injury, and its therapeutic effect is likely closely associated with the activation of the Nrf2/SLC7A11/GPX4 signaling pathway and the inhibition of ferroptosis in myocardial cells. Consequently, Calycosin, as a promising compound against doxorubicin-induced cardiotoxicity, warrants further investigation.

## Graphical Abstract

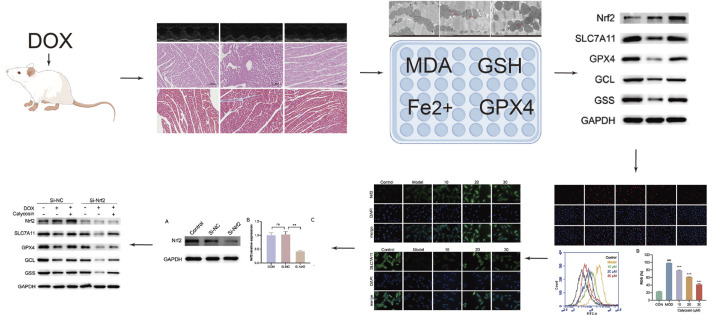

## 1 Introduction

Heart failure (HF) is a prevalent and life-threatening cardiovascular disease characterized by the heart’s inability to pump blood effectively to meet the body’s demands (Gupta et al., 2021). It represents the end-stage manifestation of several cardiovascular diseases, including coronary artery disease and hypertension (Díez and Butler, 1979; Flaherty et al., 2009). Globally, approximately 64 million individuals are affected by HF, with rising incidence and hospitalization rates, particularly among the elderly (Savarese et al., 2023). Despite significant advancements in contemporary pharmacotherapy, the management of HF remains challenging, particularly regarding disease progression and prognosis improvement (Rossignol et al., 2019). Therefore, the identification of novel treatment strategies is of paramount importance.

The pathophysiology of HF is intricate, involving oxidative stress, inflammation, and ferroptosis, among other contributing factors (Aimo et al., 2020; Xiong et al., 2024). Recent studies highlight the pivotal role of ferroptosis in the pathogenesis of HF, suggesting new therapeutic avenues (Li et al., 2024). Ferroptosis, catalyzed by ferrous ions through the Fenton reaction, results in the oxidation of polyunsaturated fatty acids in cell membranes, ultimately disrupting membrane integrity and triggering cell death (Jiang et al., 2021). Throughout the progression of HF, dysregulated iron metabolism in cardiac myocytes results in the accumulation of ferrous ions and exacerbates oxidative stress, thereby activating the ferroptosis pathway and aggravating myocardial injury and dysfunction (Jin et al., 2024; Zhang K. et al., 2023).

The Nrf2/SLC7A11/GPX4 signaling pathway is regarded as crucial for regulating ferroptosis and serves as a vital mechanism for maintaining intracellular redox balance (Dodson et al., 2019). Nuclear factor erythroid 2-related factor 2 (Nrf2) is a transcription factor that forms a complex with Kelch-like ECH-associated protein 1 (Keap1) in the cytoplasm under physiological conditions (He et al., 2020). However, under conditions of oxidative stress, Nrf2 dissociates from Keap1 and translocates to the nucleus, where it binds to antioxidant response elements (AREs) and activates the transcription of antioxidant protein genes, including Glutathione peroxidase 4 (GPX4) (Yi et al., 2024), Solute carrier family 7 member 11 (SLC7A11) (Qiu et al., 2024), glutamate-cysteine ligase (GCL) (Zhao et al., 2018), and glutathione synthetase (GSS) (Kerins and Ooi, 2017; Liu J. et al., 2023; Rojo et al., 2018). GPX4, as a glutathione peroxidase, functions in conjunction with glutathione to eliminate lipid peroxides within cells, thereby preserving membrane integrity and stability (Seiler et al., 2008). The Nrf2/SLC7A11/GPX4 signaling pathway regulates ferroptosis through two primary mechanisms: firstly, by enhancing the expression of SLC7A11, GCL, and GSS to augment glutathione (GSH) production (Yi et al., 2024); and secondly, by directly increasing GPX4 expression to enhance its reducing capacity (Yan et al., 2023). Therefore, modulation of the Nrf2/SLC7A11/GPX4 signaling pathway holds considerable promise for combating ferroptosis in cardiac myocytes.

Calycosin is a natural flavonoid compound that is prevalent in numerous traditional Chinese herbs, including Astragalus and Goji berries. Research indicates that Calycosin exerts multiple effects in cardiovascular diseases. For instance, Calycosin can reduce myocardial fibrosis and enhance cardiac function in mice following myocardial infarction (Chen et al., 2022). Calycosin also prevents oxidative stress and oxidative stress-induced apoptosis by activating aldehyde dehydrogenase 2 (Ding et al., 2023). Additionally, Calycosin inhibits foam cell formation, inflammation, and apoptosis by enhancing autophagy (Ma et al., 2022). These findings imply that Calycosin has considerable clinical implications and development prospects in the prevention and treatment of heart failure, atherosclerosis, and other cardiovascular diseases.

Furthermore, recent studies have demonstrated that Calycosin exhibits significant advantages in inhibiting ferroptosis, a form of regulated cell death associated with iron overload. Research indicates that Calycosin alleviates cerebral ischemia/reperfusion injury by inhibiting ACSL4-dependent ferroptosis. Additionally, it regulates the ferroptosis mechanism to confer protective effects in diabetic nephropathy. Recent studies also demonstrate that Calycosin modulates the expression of Nrf2, thereby exerting antioxidant effects and inhibiting apoptosis (Lu et al., 2022; Su et al., 2016). However, its protective role against ferroptosis in cardiomyocytes necessitates further investigation.

Based on these research advancements, we hypothesize that Calycosin exerts cardioprotective effects by regulating ferroptosis in cardiomyocytes. To validate this hypothesis, we will conduct both *in vivo* and *in vitro* experiments to elucidate the specific mechanisms by which Calycosin operates in heart failure. In this study, we initially assessed the therapeutic effects of Calycosin on heart failure through *in vivo* experiments. Subsequently, we conducted *in vitro* cell experiments to verify the molecular mechanisms through which Calycosin regulates ferroptosis. Finally, we further validated the targets of Calycosin in regulating ferroptosis through Nrf2 silencing. Detailed research procedures are illustrated in Graphical Abstract.

## 2 Materials and methods

### 2.1 Materials and reagents

Detection kit information is as follows: Ferrous Ion Assay Kit (Elabscience, E-BC-K773-M); MDA Assay Kit (Nanjing Jiancheng, A003-1); GPX4 ELISA Kit (Nanjing Jiancheng, H545-1-1); Microplate Method T-GSH/GSSG Assay Kit (Nanjing Jiancheng, A061-1-1); Rat NT-proBNP ELISA Kit (CUSABIO, CSB-E08752r); ROS Assay Kit (Beyotime, S0033S); BCA Protein Assay Kit (CWBIO, CW0014). Antibody information is as follows: Nrf2 Recombinant Antibody (proteintech, 80593-1-RR); Anti-xCT Antibody [EPR27115-64] (abcam, AB307601); GPX4 Monoclonal Antibody (proteintech, 67763-1-Ig); GSS Polyclonal Antibody (proteintech, 15712-1-AP); GCLC Polyclonal Antibody (proteintech, 12601-1-AP); GAPDH Rabbit pAb (proteintech, 10,494). Other reagent information is as follows: Calycosin (Yuanye, 20,575-57-9, HPLC ≥ 98%); Doxorubicin (VETEC, 53123-88-9); PI (Propidium Iodide) Staining Solution (Solarbio, C0080); CCK-8 Solution (Vazyme, A311-02-AA); ECL Substrate A and Peroxide Solution B (Vazyme, E412-02); DAPI Staining Solution (Beyotime, C1005); Anti-fade Mounting Medium (Solarbio, S2100); FBS (BasalMedia, S660JJ); DMEM (BasalMedia, L110KJ); CCK-8 Solution (Vazyme, A311-02-AA); DMSO (Servicebio, GC203002-100 mL); Paraformaldehyde (Servicebio, G1101-500ML), Electron microscope fixative (Servicebio, G1102-100ML), PBS (Servicebio, G4202-500ML).

### 2.2 Animal models and treatment

Thirty 8-week-old Wistar rats, with an average weight of approximately 200 ± 20 g, were utilized for the experiment. The rats were procured from Beijing Vital River Laboratory Animal Technology Co., Ltd. All rats were housed in a controlled environment at the Animal Experiment Center of the Affiliated Hospital of Shandong University of Traditional Chinese Medicine. The housing conditions were maintained at a temperature of 22°C ± 2°C and a humidity of 50%–60%. The rats were provided with unrestricted access to water and standard feed to promote normal growth and development. To minimize the influence of environmental factors on the experimental outcomes, the rats were acclimated for 1 week prior to the commencement of the experiment.

At the outset of the experiment, the thirty rats were randomly assigned to three groups: the control group (CON), the model group (MOD), and the Calycosin group (CAL). Intraperitoneal injections of doxorubicin were utilized to establish a heart failure model in this study. Doxorubicin is frequently employed to induce experimental heart failure owing to its cardiotoxic effects (Abu and El-Magd, 2018). Specifically, doxorubicin was dissolved in saline and administered at a dosage of 4 mg/kg body weight once weekly for four consecutive weeks to establish the model (Chen et al., 2024). The control group received equivalent volumes of saline injections as a placebo. Following the establishment of the model, the rats exhibited characteristic signs of heart failure, including weight loss, reduced activity, and tachypnea. To confirm the successful establishment of the heart failure model, echocardiography was employed to evaluate the cardiac function of the rats.

Following the successful establishment of the model, the rats in the Calycosin group underwent treatment with Calycosin. Calycosin was dissolved in saline containing dimethyl sulfoxide (DMSO) and sulfobutylether-β-cyclodextrin (SBE-β-CD) and administered via intraperitoneal injection at a dosage of 10 mg/kg once daily for four consecutive weeks (Liu H. et al., 2023). The remaining groups continued to receive equivalent volumes of saline containing DMSO and SBE-β-CD. All experimental procedures were formally approved by the Ethics Committee of the Affiliated Hospital of Shandong University of Traditional Chinese Medicine and strictly adhered to the ethical standards established in the “Guidelines for Animal Experiments of the Chinese Medical Ethics Committee.” The ethics approval number is AWE-2023-009.

### 2.3 Cardiac ultrasound

To obtain ultrasound images of the rat heart, the rats were anesthetized using pentobarbital sodium to ensure immobility. The chest hair of the rats was shaved, and ultrasound gel was applied to facilitate sound wave conduction. A high-frequency ultrasound probe was placed on the left side of the rat’s chest, and the left ventricle was identified using B-mode. Subsequently, M-mode was selected to assess the cardiac function of the rat.

### 2.4 Histological staining

After anesthetizing the rats, the hearts are extracted and rinsed with PBS to clean them. The left ventricular myocardial tissue was then isolated for pathological examination. The heart tissue is then fixed with 4% paraformaldehyde, followed by a dehydration process. Next, the heart tissue is embedded in paraffin and sectioned into 4-micron slices using a microtome (El-Magd et al., 2017). The sections are mounted on glass slides and undergo deparaffinization and rehydration. Subsequently, hematoxylin and eosin (H&E) staining and Masson’s trichrome staining are performed. The stained sections are scanned using an optical scanner. Finally, the sections are subjected to pathological analysis, and ImageJ software is used to analyze the Masson staining results.

### 2.5 Transmission electron microscopy

As previously described, the isolated left ventricular myocardial tissue was placed on a pre-cooled workbench and washed with pre-cooled PBS. The myocardial tissue was cut into 0.5 mm thick slices and fixed using pre-cooled electron microscope fixative (Solarbio, P1126). After fixation, dehydration was performed, and the tissue was embedded in epoxy resin. Once the resin had hardened, an ultra-thin slicer was used to cut the samples into 70 nm ultra-thin sections. After the sections were stained with uranium and lead, they were observed using a transmission electron microscope, and images of the cardiac ultrastructure were captured for analysis.

### 2.6 Detection of ferrous ions, GSH/GSSH, MDA, GPX4, and NT-Pro BNP

After the rat myocardial tissue was isolated, ferrous ions, GSH/GSSH, MDA, and GPX4 were detected using appropriate test kits. According to the instructions, absorbance values were recorded at wavelengths of 590 nm, 532 nm, 450 nm, and 405 nm, respectively. Blood was collected from the rat’s abdominal aorta and allowed to stand for 30 min before being centrifuged at 3,000 rpm for 10 min. The supernatant was then used for testing. NT-Pro BNP levels in rat serum were measured using an NT-Pro BNP test kit, and the absorbance was recorded at 450 nm according to the kit’s instructions. To ensure the credibility and reproducibility of the experimental results, five samples were taken from each group for repetition in the above experiments.

### 2.7 Cell models and treatment

Rat H9c2 cells were purchased from the Chinese Academy of Sciences Cell Bank (Beijing, China). The H9C2 cells were removed from the liquid nitrogen and thawed quickly in a 37°C water bath. The thawed cells were then transferred to DMEM containing 10% fetal bovine serum (FBS) and 1% penicillin and streptomycin, and cultured at 37°C with 5% CO_2_ and 95% air until they reached 70%–80% confluence for passaging. After passaging, cells were allowed to grow to an appropriate confluence before performing experiments. The cells were divided into three groups: the control group, the model group, and the Calycosin group. The model group and Calycosin group were incubated with 0.1 μM doxorubicin solution for 24 h to establish a damage model, while the control group was treated with an equal volume of PBS (Zhang X. J. et al., 2023). After incubation for 24 h, the cells were treated with different concentrations of mullein isoflavone solution (0 μM, 10 μM, 20 μM, 30 μM, 50 μM), and the control group and model group were treated with the same amount of DMSO. After that, incubate for 24 h. Subsequently, cells from each group were collected for further analysis.

### 2.8 si-Nrf2

For the si-Nrf2 experiment, cells were first seeded in a 6-well plate and allowed to grow to 60%–70% confluence. A transfection mixture was prepared by combining siRNA targeting Nrf2 with a transfection reagent according to the manufacturer’s guidelines. This mixture was added to the cells, and incubation was carried out for 24–48 h to facilitate uptake. After incubation, the cells were harvested, and Nrf2 knockdown was analyzed using Western blotting.

### 2.9 CCK8

To each well of a 96-well plate, 100 μL of H9C2 cardiomyocyte suspension was added and incubated at 37°C with 5% CO2 for 24 h to allow the cells to adhere. Next, doxorubicin was introduced at a final concentration of 0.1 μM to establish the damage model, while the control group received an equal volume of PBS. Incubation was carried out for 24 h. Following this, varying concentrations of Calycosin (0 μM, 10 μM, 20 μM, 30 μM, 50 μM) were added, and incubation continued for an additional 24 h. After treatment, 10 μL of CCK-8 Solution was added to each well, mixed gently, and incubated in the incubator for 2 h. The absorbance (OD value) at 450 nm was measured using a microplate reader. The protective effect of the drug against doxorubicin-induced damage in H9C2 cells was evaluated by calculating cell viability.

### 2.10 ROS detection

To assess cellular ROS levels, a ROS detection kit was used. H9C2 cells were seeded into a 6-well plate and cultured until they reached 70%–80% confluence. After treatment, the cell suspension was transferred to EP tubes, centrifuged at 1,000 rpm for 5 min, and the supernatant was discarded. DCFH-DA was then diluted at a 1:1,000 ratio in serum-free medium to a final concentration of 10 μM, and 1 mL of the diluted solution was added to each tube. The tubes were incubated at 37°C for 20 min to allow DCFH-DA to enter the cells. Following incubation, the cells were washed twice with serum-free medium, centrifuging at 1,000 rpm for 5 min each time. Finally, each tube was resuspended in 200 μL of serum-free cell culture medium and analyzed using a flow cytometer.

### 2.11 PI staining

For PI staining, after the cell culture medium was removed from the 24-well plate, 1 mL of methanol was added to each well for 20 min to fix the cells. After fixation, the methanol was discarded, and the cells were washed twice with 2 × SSC (0.3 M NaCl, 0.03 M sodium citrate, pH 7.0) for 2 min each. The cells were then treated with a permeabilization solution for 10 min, followed by two washes with 2 × SSC solution for 2 min each. DAPI staining solution was added next, and the cells were incubated for 5 min before being washed twice with 2 × SSC solution for 2 min each. A 500 nM PI working solution was prepared by diluting a 1 mg/mL PI stock solution 1:3,000 in 2 × SSC. Then, 300 μL of this solution was added to each well, and the cells were incubated for 5 min. Afterward, the cells were washed twice with 2 × SSC solution for 2 min each, followed by the addition of an anti-fade mounting medium. Images were captured using a fluorescence microscope.

### 2.12 Immunofluorescence

Following the PI staining procedure, the cells were fixed and permeabilized. Then, 200 µL of a 5% BSA blocking solution was added to each well and incubated at room temperature for 1 h. After blocking, the cells were washed twice with PBS. Next, 200 µL of the primary antibody solution (including Nrf2 Recombinant Antibody, Anti-xCT Antibody [EPR27115-64], and GPX4 Monoclonal Antibody) was added to each well and incubated overnight at 4°C. After incubation, the cells were washed again with PBS. Subsequently, 200 µL of the secondary antibody solution was added to each well and incubated at room temperature for 90 min. Following the secondary antibody incubation, the cells were washed again. Finally, 200 µL of DAPI staining solution was added to each well and incubated at room temperature for 10 min. After the final incubation, an anti-fade mounting medium was added, and fluorescence microscopy was performed for detection.

### 2.13 Western blotting (WB)

Extract proteins from cardiac tissues and cells using a total protein extraction kit. Determine the protein concentration using a BCA protein assay kit, and dilute with loading buffer as needed to ensure equal protein concentrations across samples. Next, separate the proteins by electrophoresis using SDS-PAGE and transfer the proteins to a PVDF membrane. Block the membrane by incubating it in a 5% skim milk solution at room temperature for 1 h to prevent non-specific binding. After blocking, treat the membrane with the appropriate concentration of primary antibody solution and incubate overnight at 4°C. Following this, wash the membrane with PBS to remove unbound primary antibodies. Incubate the membrane with a secondary antibody solution conjugated with horseradish peroxidase at room temperature for 1 h to ensure complex formation between the primary and secondary antibodies. Wash the membrane again with PBS to remove excess secondary antibody. Finally, prepare the ECL detection solution by mixing ECL Substrate A (Vazyme, E412-02) with Peroxide Solution B (Vazyme, E412-02), incubate in the dark for 3 min, and perform imaging. Analyze the band density using ImageJ software, normalizing to GAPDH as an internal control. Calculate the expression level of the target protein by determining the ratio of the target protein band density to the internal control band density.

### 2.14 Statistical analysis

We used IBM SPSS Statistics 27 and GraphPad Prism 9 for statistical analysis and visualization. The data for each experimental group are presented as the mean ± standard deviation (mean ± SD) from at least three independent replicates. We employed SPSS to test the normality of the data. For data that followed a normal distribution, independent samples t-test was used to analyze the differences in means between groups; for data that did not follow a normal distribution, logarithmic or square root transformations were applied to make the data closer to a normal distribution before comparing group differences. A *p*-value of less than 0.05 was considered statistically significant.

## 3 Results

### 3.1 Calycosin improved heart function and reduced myocardial damage in rats with heart failure

**FIGURE 1:**
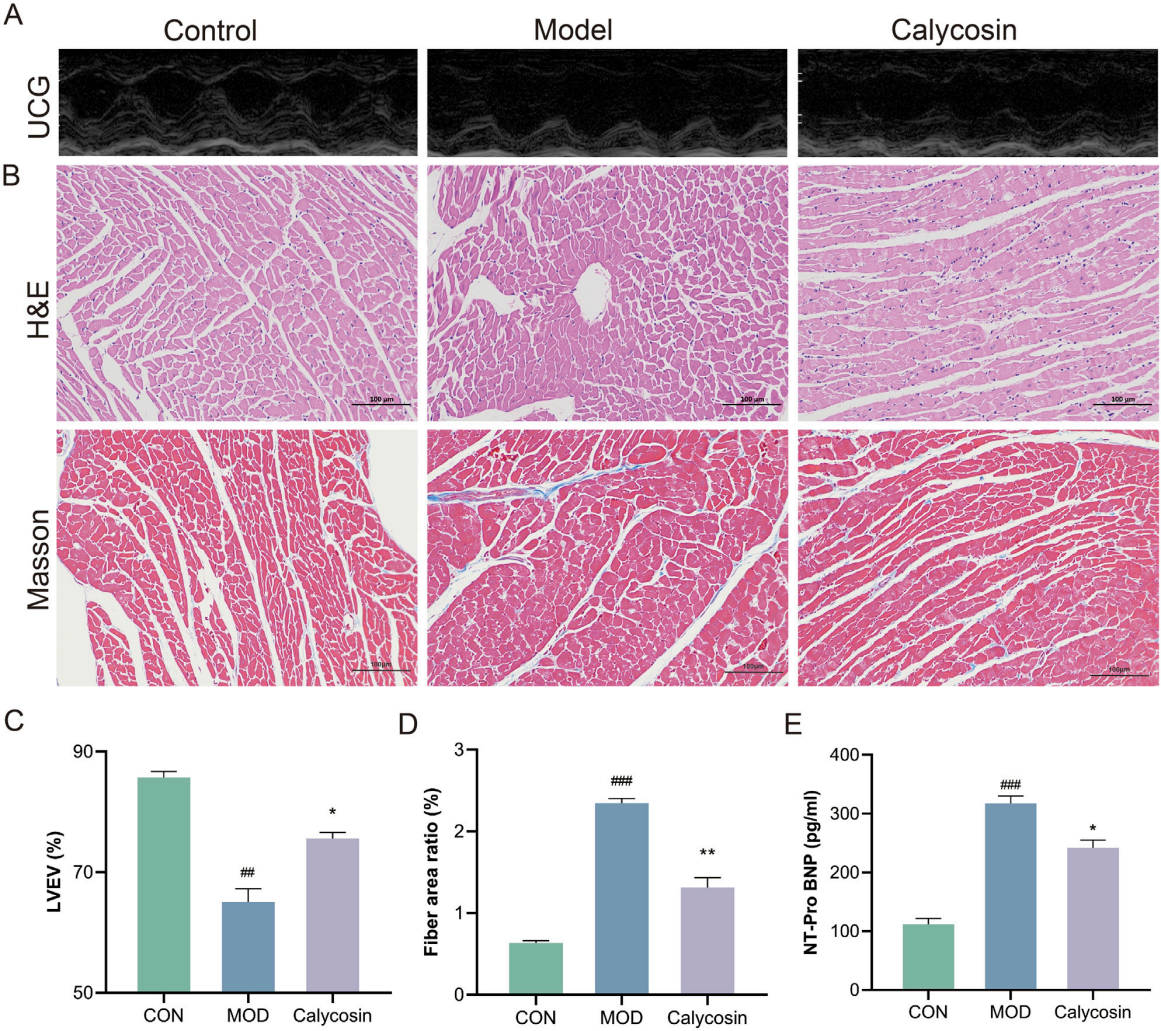
**(A)** Representative cardiac ultrasound images of each group (×200); **(B)** represents the representative image of H&E and Masson staining (×200); **(C)** represents LVEF level in each group (N = 5); **(D)** represents the proportion of myocardial fibrosis in each group (N = 3); **(E)** represents the level of NT-Pro BNP in each group (N = 3). Where ## means *p* < 0.01 compared with the control group, and ### means *p* < 0.001 compared with the control group; * means *p* < 0.05 compared with the model group, ** means *p* < 0.01 compared with the model group. Data are presented as mean ± standard deviation (mean ± SD).

To evaluate the therapeutic effect of Calycosin on heart failure in rats, cardiac ultrasound examinations, NT-Pro BNP assays, and cardiac tissue pathology analyses were conducted. As illustrated in Figure 1, Calycosin significantly improved cardiac function in rats with heart failure. Notably, in the cardiac ultrasound examination, the left ventricular ejection fraction (LVEF) of rats treated with Calycosin was significantly increased (*p* < 0.05), indicating a substantial enhancement in cardiac function. Additionally, Calycosin significantly reduced NT-Pro BNP levels (*p* < 0.05), further corroborating its beneficial effects in the treatment of heart failure. Pathological analysis demonstrated that Calycosin intervention markedly alleviated myocardial cell damage, as evidenced by effective repair of myocardial cell structure, improved myocardial cell integrity, and a significant reduction in the area of myocardial fibrosis (*p* < 0.05). These results suggest that Calycosin not only improves cardiac function but also mitigates myocardial cell damage and fibrosis, thereby contributing positively to the treatment of heart failure.

### 3.2 Calycosin decreased the level of ferroptosis in heart tissue of rats with heart failure

**FIGURE 2:**
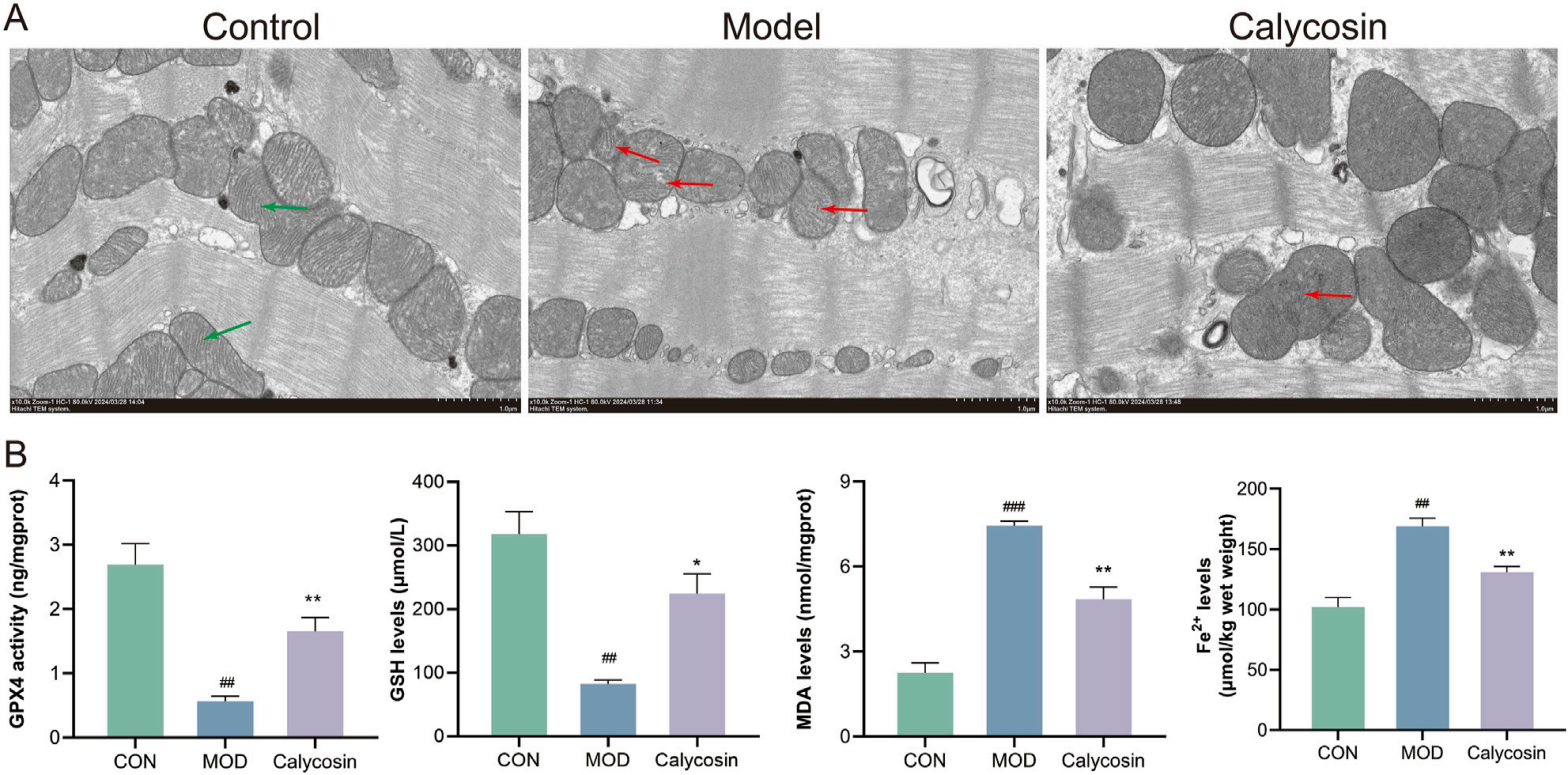
**(A)** shows the representative image of transmission electron microscope; **(B)** is the expression level of ferroptosis markers (N = 3). Where ## means *p* < 0.01 compared with the control group, and ### means *p* < 0.001 compared with the control group; * means *p* < 0.05 compared with the model group, ** means *p* < 0.01 compared with the model group. Data are presented as mean ± standard deviation (mean ± SD).

To investigate the impact of Calycosin on ferroptosis in myocardial tissue of heart failure rats, TEM was conducted to observe mitochondrial morphology and analyze its regulatory effects on ferroptosis. Additionally, ferroptosis markers in the myocardial tissue were measured, including ferrous ions, GSH, MDA, and GPX4 activity. TEM results (Figure 2A) indicated that in the control group, the mitochondrial membrane structure was intact, with orderly mitochondrial cristae (green arrows) and a regular shape. In the model group, mitochondrial cristae were fractured and diminished (red arrows), with partial membrane rupture and mitochondrial swelling. In the Calycosin group, mitochondrial damage was ameliorated, with decreased cristae fracture and a relatively intact membrane structure. Malondialdehyde, a final product of lipid peroxidation, reflects the degree of membrane phospholipid oxidation. Results (Figure 2B) indicated that Calycosin significantly reduced MDA levels (*p* < 0.01), suggesting its effective role in mitigating lipid peroxidation. The increase in ferrous ions is another key feature of ferroptosis, as it exacerbates cellular damage by facilitating the Fenton reaction, leading to the generation of reactive oxygen species. The ferrous ion levels in the Calycosin-treated group were significantly lower than those in the control group (*p* < 0.01), demonstrating its beneficial effect on stabilizing iron ions (Figure 2B). Glutathione, a major intracellular antioxidant, is associated with ferroptosis when its levels decrease. Calycosin treatment elevated GSH levels (*p* < 0.05), indicating its role in promoting oxidative stress defense (Figure 2B). Glutathione peroxidase 4 (GPX4) is a critical antioxidant enzyme that catalyzes the reduction of lipid peroxides, thereby preventing cellular damage. Calycosin significantly increased GPX4 activity (*p* < 0.01), further confirming its potential role in combating ferroptosis (Figure 2B).

### 3.3 Calycosin increased the expression of Nrf2/SLC7A11/GPX4 pathway protein

**FIGURE 3:**
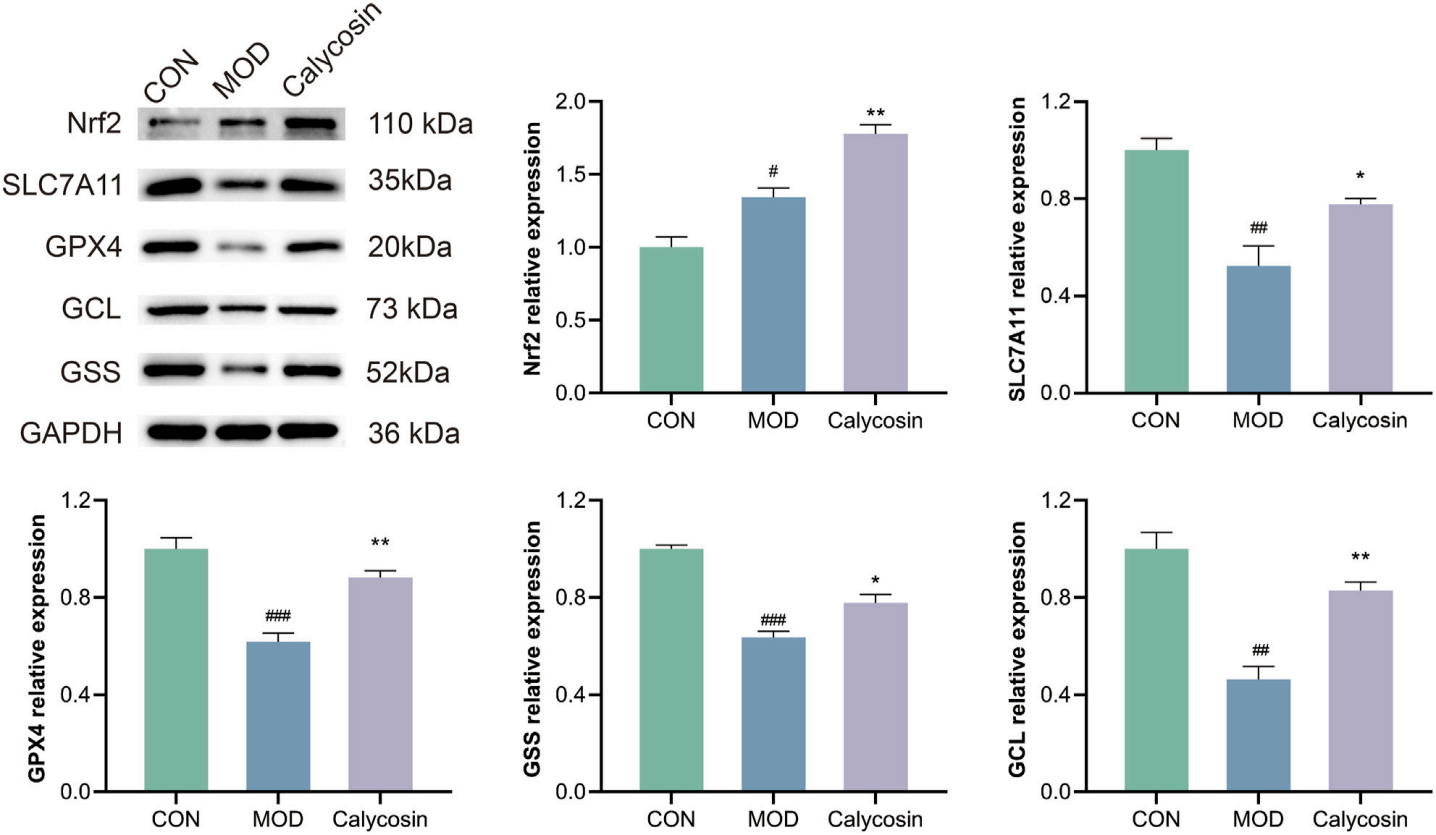
WB representative images of pathway proteins in cardiac tissue of rats with heart failure after Calycosin intervention and relative expression levels of each protein (N = 3). Where # means *p* < 0.05 compared with the control group, ## means *p* < 0.01 compared with the control group, and ### means *p* < 0.001 compared with the control group; * means *p* < 0.05 compared with the model group, ** means *p* < 0.01 compared with the model group. Data are presented as mean ± standard deviation (mean ± SD).

To validate the regulatory effects of Calycosin on the Nrf2/SLC7A11/GPX4 pathway proteins, Western blot (WB) analysis was performed. As illustrated in Figure 3, Calycosin treatment significantly upregulated the expression of the antioxidant protein Nrf2 (*p* < 0.01), as well as the levels of its downstream antioxidant and ferroptosis-related proteins. This finding indicates that Calycosin positively regulates the expression of Nrf2/SLC7A11/GPX4 pathway proteins, further corroborating its antioxidant and anti-ferroptosis capabilities.

### 3.4 Calycosin alleviates myocardial cell damage

**FIGURE 4:**
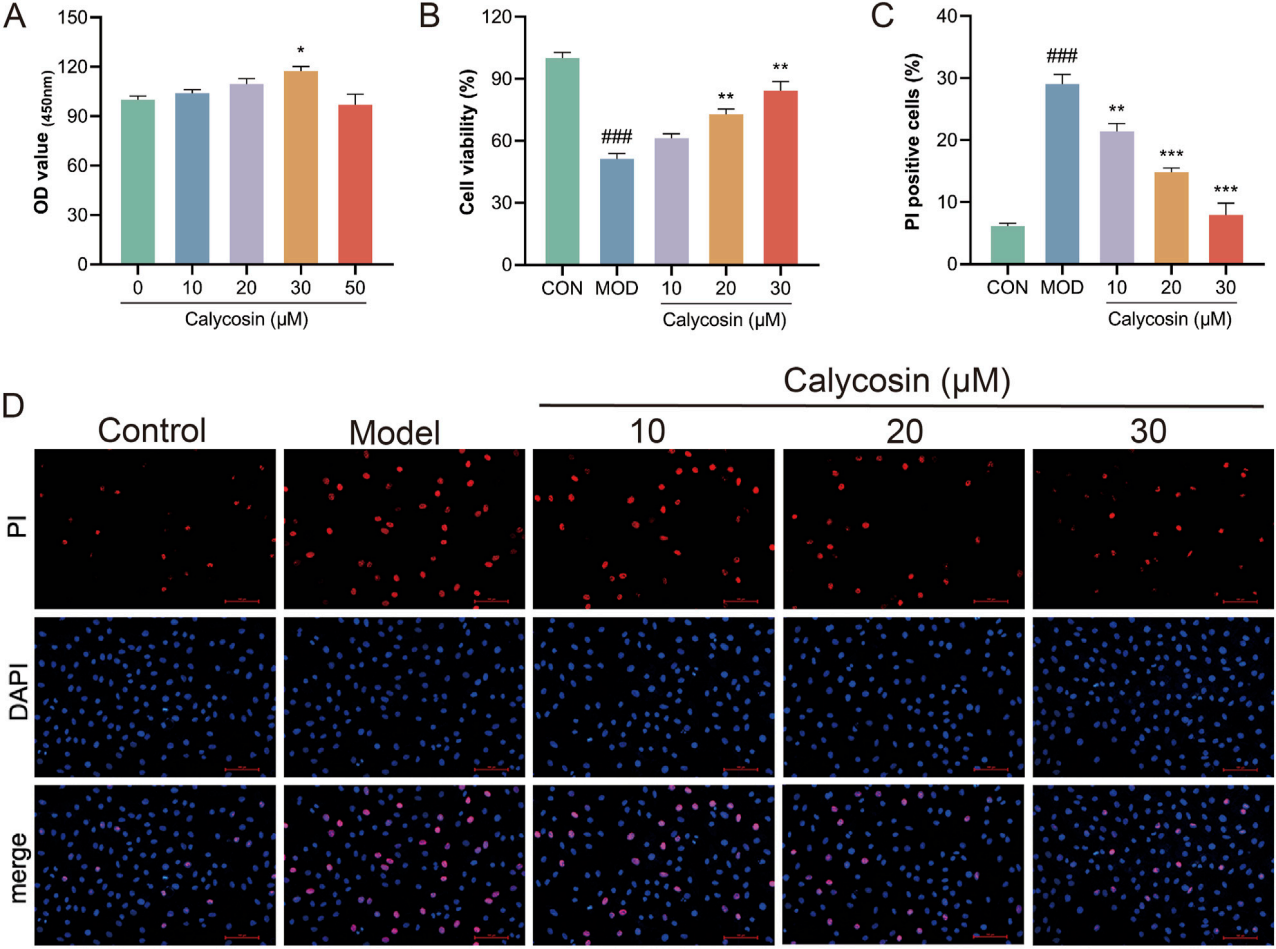
**(A)** shows the effect of Calycosin on cell viability at different concentrations (N = 3); **(B)** is the effect of Calycosin on cell activity after doxorubicin intervention (N = 3); **(C)** is the result of PI staining in each group (N = 3); **(D)** represents the representative images of each group stained by PI. Where ### means *p* < 0.001 compared with the control group; ** means *p* < 0.01 compared with the model group, *** means *p* < 0.001 compared with the model group. Data are presented as mean ± standard deviation (mean ± SD).

To further validate the protective effects of Calycosin and its potential mechanisms, *in vitro* cell experiments were conducted to determine the optimal intervention concentration of Calycosin by treating cells with various concentrations. CCK-8 assay results (Figure 4A) indicated that Calycosin significantly protected cells from damage at lower concentrations (10–30 μM) while exhibiting cytotoxicity at higher concentrations (50 μM), consistent with the toxic effects observed for other plant compounds at elevated concentrations. To evaluate the protective effects of Calycosin on cardiomyocytes, cells were treated with doxorubicin and Calycosin, and the protective effects were assessed using CCK-8 and propidium iodide (PI) staining. Results (Figures 4B–D) demonstrated that Calycosin effectively mitigated doxorubicin-induced cell damage, and this protective effect was concentration-dependent. At a concentration of 30 μM, Calycosin exhibited the most significant protective effect (*p* < 0.01).

### 3.5 Calycosin improves the antioxidant capacity of H9c2

**FIGURE 5:**
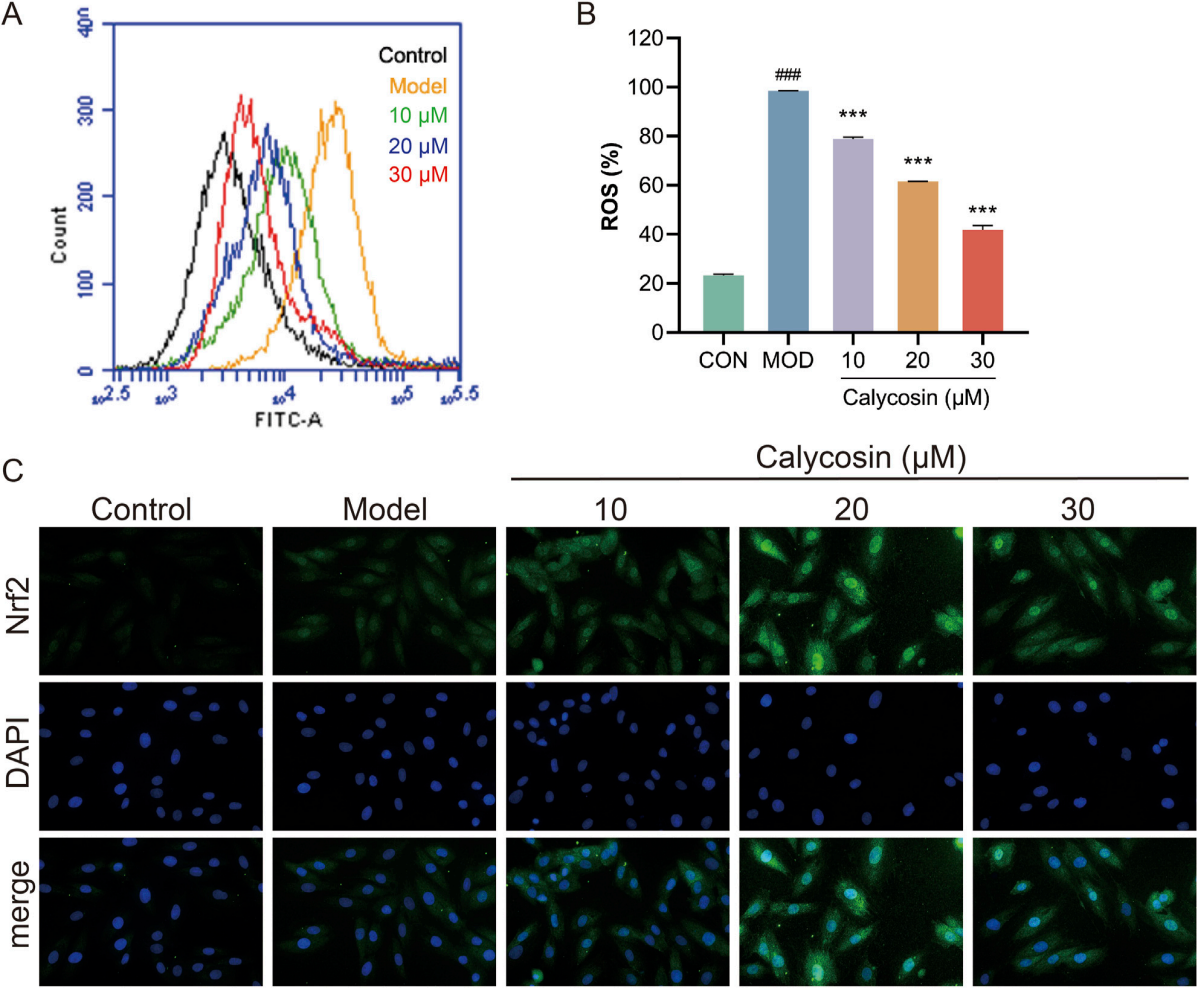
**(A)** shows the images of reactive oxygen species detection in each group; **(B)** is the level of reactive oxygen species in each group (N = 3); **(C)** is the representative image of Nrf2 immunofluorescence. Where ### means *p* < 0.001 compared with the control group; *** means *p* < 0.001 compared with the model group. Data are presented as mean ± standard deviation (mean ± SD).

Ferroptosis is a form of cell death resulting from the oxidation of polyunsaturated fatty acids in the cell membrane, catalyzed by reactive oxygen species (ROS) and ferrous ions through the Fenton reaction, which leads to damage of the cell membrane structure. Consequently, the levels of ROS and the extent of oxidative stress play a crucial role in sensitivity to ferroptosis. To evaluate the protective effects of Calycosin against cellular oxidative stress, intracellular ROS levels were measured. Results (Figures 5A, B) indicated that Calycosin significantly mitigated cell damage induced by doxorubicin treatment. Furthermore, results from NRF2 immunofluorescence staining confirmed that Calycosin promotes the translocation of NRF2 to the cell nucleus (Figure 5C). As a critical antioxidant transcription factor, the activation of NRF2 can upregulate the expression of various antioxidant genes, thereby effectively counteracting cell damage induced by oxidative stress.

### 3.6 Calycosin decreased the ferroptosis level of H9c2 cardiomyocytes

**FIGURE 6:**
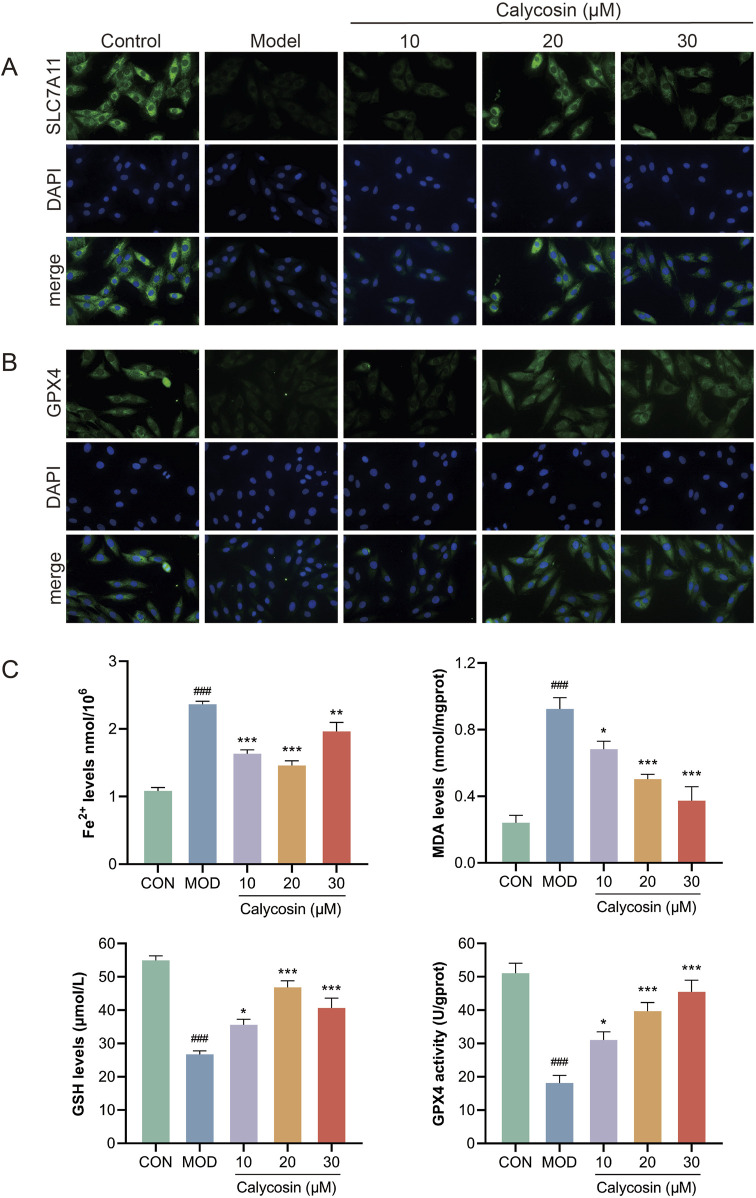
**(A)** shows the representative image of SLC7A11 immunofluorescence; **(B)** is the representative image of GPX4 immunofluorescence; **(C)** was the level of ferroptosis marker in each group (N = 3). Where # means *p* < 0.05 compared with the control group, ## means *p* < 0.01 compared with the control group, and ### means *p* < 0.001 compared with the control group; * means *p* < 0.05 compared with the model group, ** means *p* < 0.01 compared with the model group, and *** means *p* < 0.001 compared with the control group. Data are presented as mean ± standard deviation (mean ± SD).

To evaluate the ferroptosis-inhibiting effects of Calycosin, four ferroptosis markers were measured in the cells: MDA, ferrous ions, GSH, and GPX4 activity. Results (Figure 6C) indicated that Calycosin significantly reduced MDA levels, suggesting its effective role in mitigating lipid peroxidation. The ferrous ion levels in the Calycosin-treated group were significantly lower than those in the model group, demonstrating its beneficial role in stabilizing iron ions. Additionally, Calycosin treatment elevated GSH levels, indicating its role in enhancing oxidative stress defense. Moreover, Calycosin significantly enhanced GPX4 activity, further corroborating its potential in combating ferroptosis. Additionally, immunofluorescence staining was performed for ferroptosis-related proteins SLC7A11 and GPX4. SLC7A11, an important antioxidant transporter protein involved in the transport of glutamate and cysteine, plays a crucial role in maintaining oxidative balance. Calycosin treatment significantly upregulated SLC7A11 expression, suggesting that it may combat ferroptosis by promoting the synthesis of intracellular antioxidant substances (Figure 6A). The immunofluorescence results for GPX4 also demonstrated a significant increase in expression in the Calycosin-treated group, consistent with its protective role in ferroptosis (Figure 6B).

### 3.7 Calycosin upregulates Nrf2/SLC7A11/GPX4 signaling pathway protein

**FIGURE 7:**
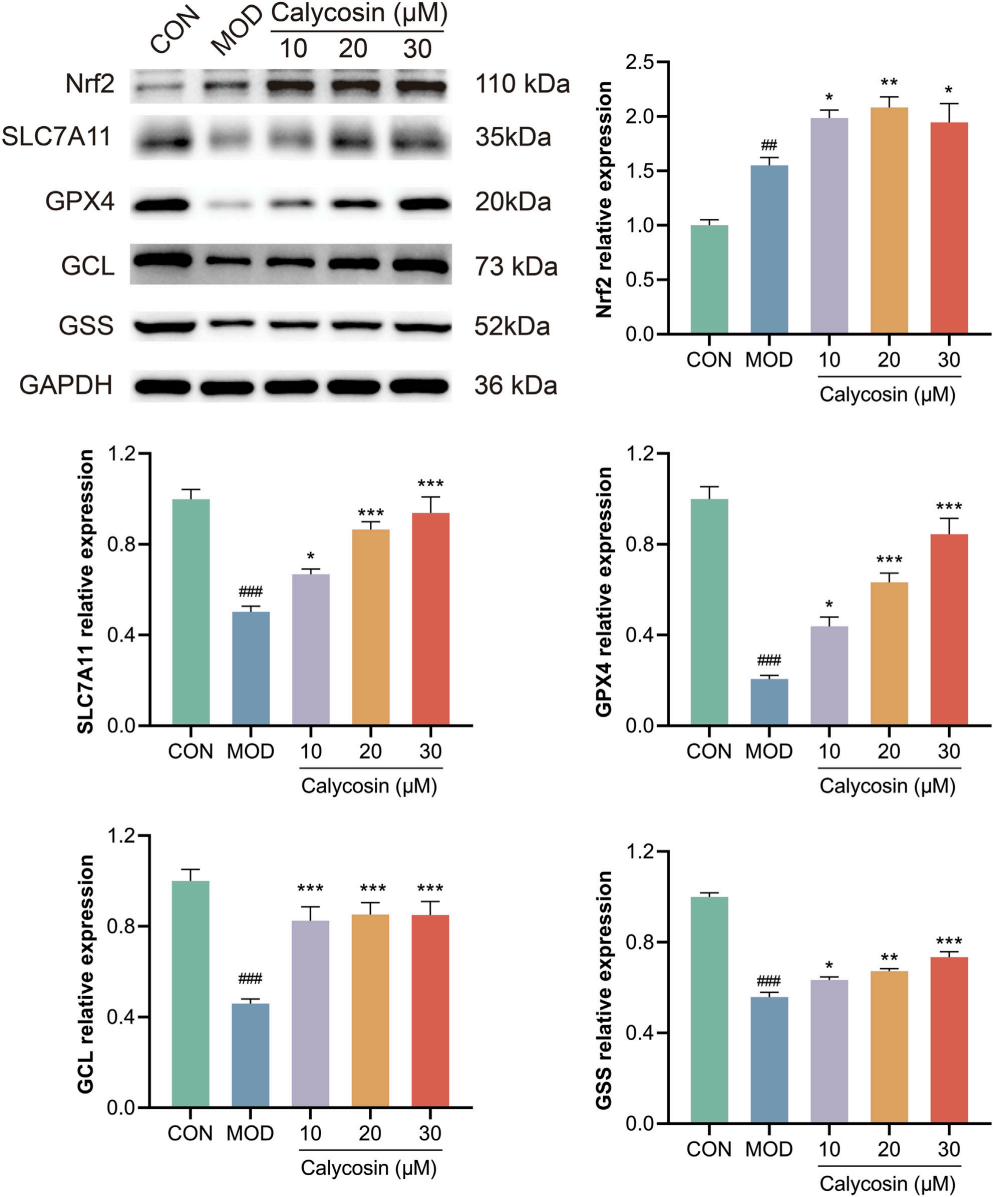
WB representative images and relative expression levels of related proteins (N = 3). Where # means *p* < 0.05 compared with the control group, ## means *p* < 0.01 compared with the control group, and ### means *p* < 0.001 compared with the control group; * means *p* < 0.05 compared with the model group, ** means *p* < 0.01 compared with the model group, and *** means *p* < 0.001 compared with the control group. Data are presented as mean ± standard deviation (mean ± SD).

To assess the regulatory effects of Calycosin on the Nrf2/SLC7A11/GPX4 signaling pathway, Western blot analysis was employed to detect the expression of key pathway proteins, including Nrf2, SLC7A11, GPX4, GSS, and GCL. Results (Figure 7) indicated that Calycosin treatment significantly upregulated the expression levels of Nrf2, SLC7A11, and GPX4, and increased the levels of GSS and GCL. These proteins serve as crucial regulatory factors in the ferroptosis process. Specifically, the upregulation of Nrf2, an important antioxidant transcription factor, suggests that Calycosin may enhance cellular antioxidant capacity by activating Nrf2. The increase in SLC7A11 and GPX4 indicates that Calycosin enhances the functionality of the antioxidant system, thereby mitigating the effects of ferroptosis. Additionally, the upregulation of GSS and GCL further supports the notion that Calycosin enhances glutathione synthesis and maintains intracellular oxidative balance.

### 3.8 The protective effect of Calycosin on cardiomyocytes was cancelled by silencing Nrf2

**FIGURE 8:**
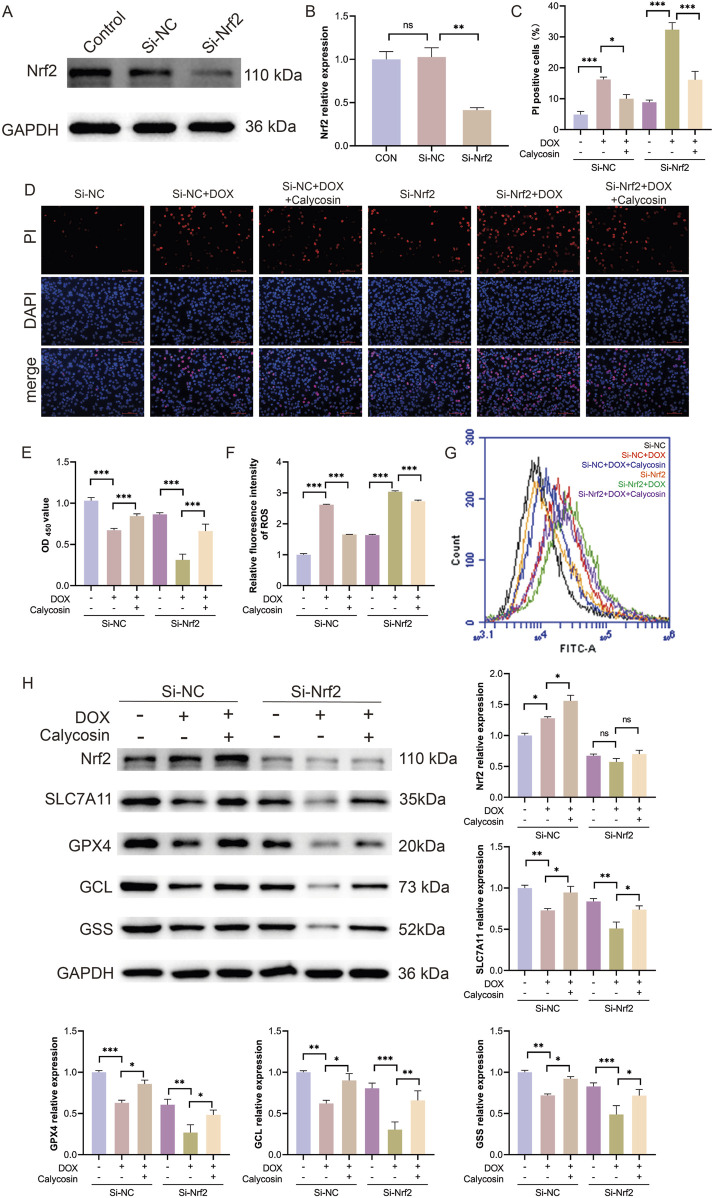
**(A)** shows the representative WB image of si-Nrf2 silencing efficiency; **(B)** is the relative expression level of Nrf2 (N = 3); **(C)** was the ratio of PI positive cells in each group (N = 3); **(D)** is the representative image stained by PI; **(E)** was the CCK8 result of cell activity in each group (N = 3); **(F)** is the level of reactive oxygen species in each group (N = 3); **(G)** represents reactive oxygen species images of each group; **(H)** represents WB representative images and relative expression levels of each protein in each group (N = 3). Where * means *p* < 0.05, ** means *p* < 0.01, and *** means *p* < 0.001. Data are presented as mean ± standard deviation (mean ± SD).

To further validate the mechanism of Calycosin, small interfering RNA (siRNA) technology was employed to silence the Nrf2 gene and reduce its expression levels. Initially, Western blot analysis was used to confirm the efficiency of Nrf2 silencing. Results (Figures 8A, B) indicated a significant reduction in Nrf2 expression (*p* < 0.01), confirming the successful silencing of the Nrf2 gene. To evaluate the protective effects of Calycosin under Nrf2 silencing conditions, CCK-8 and PI staining experiments were conducted (Figures 8C–E). CCK-8 results indicated that, following Nrf2 silencing, Calycosin’s protective effect against doxorubicin-induced cell damage was significantly diminished. PI staining further confirmed an increase in cell death rates, suggesting that Nrf2 silencing markedly reduced the cell protection efficacy of Calycosin. Additionally, intracellular ROS levels were measured. Results (Figures 8F, G) indicated that under Nrf2 silencing conditions, ROS levels were significantly elevated, indicating an enhancement of ferroptosis-related oxidative stress. This sharply contrasts with the effective reduction of ROS levels by Calycosin under normal conditions, further supporting the critical role of Nrf2 in its antioxidant effects. To explore the role of the Nrf2/SLC7A11/GPX4 signaling pathway in Calycosin’s anti-ferroptosis effects, the expression of pathway-related proteins, including Nrf2, SLC7A11, GPX4, GCL, and GSS, was assessed. Results (Figure 8H) demonstrated that Nrf2 silencing significantly decreased the expression levels of its downstream proteins, SLC7A11, GPX4, GCL, and GSS. This indicates that Nrf2 silencing reversed the anti-ferroptosis effects of Calycosin mediated by the Nrf2/SLC7A11/GPX4 signaling pathway. In summary, Nrf2 silencing significantly weakened Calycosin’s protective effects on cells and was associated with a notable increase in ROS levels. These results further demonstrate that Calycosin combats ferroptosis by regulating the Nrf2/SLC7A11/GPX4 signaling pathway, highlighting the central role of Nrf2 in Calycosin-mediated ferroptosis inhibition.

## 4 Discussion

Over the past decade, ferroptosis—a novel form of programmed cell death—has been demonstrated to play a significant role in various pathophysiological conditions, including heart failure (Fang et al., 2023). However, the specific mechanisms underlying ferroptosis in heart failure and the corresponding effective intervention strategies have yet to be fully elucidated (Yang et al., 2022). In this study, we systematically explored, for the first time, the mechanism by which calycosin alleviates heart failure by inhibiting ferroptosis through the regulation of the Nrf2/SLC7A11/GPX4 signaling pathway.

Heart failure, a complex cardiovascular disease, currently lacks effective treatment options, particularly regarding the inhibition of cardiomyocyte death (Chen et al., 2023; Joseph et al., 2023). Ferroptosis, triggered by intracellular iron accumulation and lipid peroxidation, has not been adequately studied within the context of heart failure (Del et al., 2019). Existing research indicates that Nrf2, a key transcription factor in the cellular antioxidant stress response, can mitigate oxidative stress and ferroptosis by regulating downstream genes (Yan et al., 2023). Therefore, exploring the role of Nrf2 in heart failure and its potential therapeutic strategies for regulating ferroptosis is of considerable significance.

Previous studies have demonstrated that Calycosin exerts therapeutic effects on heart failure through various mechanisms, including the modulation of autophagy, alleviation of inflammation, and reduction of myocardial fibrosis (Lu et al., 2021; Wang et al., 2022). Additionally, Calycosin has been shown to inhibit ferroptosis in models of brain ischemia-reperfusion and renal injury (Liu H. et al., 2023; Huang et al., 2022). However, its effects on ferroptosis in heart failure have yet to be systematically investigated. This research found that the administration of Calycosin significantly improved doxorubicin-induced heart failure, evidenced by an enhanced ejection fraction and reduced serum NT-Pro BNP levels. Simultaneously, Calycosin alleviates myocardial tissue damage, including the repair of myocardial cell injury and a reduction in myocardial fibrosis. These findings indicate that Calycosin has a significant therapeutic effect on heart failure and offers protection to myocardial cells. Furthermore, similar results were obtained from *in vitro* cell experiments, wherein Calycosin improved doxorubicin-induced cell damage, further validating its protective effect on myocardial cells.

Ferroptosis is a form of programmed cell death characterized by the Fenton reaction, in which polyunsaturated fatty acids in the cell membrane react with reactive oxygen species, catalyzed by ferrous ions (Dixon and Olzmann, 2024). This process results in damage to the cell membrane and ultimately leads to cell death. Consequently, specific molecules are utilized as markers for assessing ferroptosis levels. Malondialdehyde (MDA), a product of lipid peroxidation, is typically associated with the attack of free radicals on lipid molecules, such as phospholipids in the cell membrane (Zhang X. et al., 2023). Measuring MDA levels can assess the extent of membrane lipid peroxidation and oxidative damage, with elevated MDA levels often indicating exacerbated ferroptosis. The concentration of ferrous ions, which act as catalysts, is closely related to the sensitivity of cells to ferroptosis. Glutathione (GSH), a major intracellular reductant, acts as a substrate for glutathione peroxidase 4 (GPX4) and collaborates with GPX4 to exert antioxidant effects (Ursini and Maiorino, 2020). Reactive oxygen species (ROS), significant oxidants, directly participate in the oxidation of membrane phospholipids. Therefore, measuring the levels of these molecules can evaluate the degree of ferroptosis. Our experimental results indicate that following Calycosin intervention, levels of MDA and ferrous ions are significantly reduced, while GSH and GPX4 activities within the antioxidant system are significantly increased. These findings suggest that Calycosin can reduce the extent of ferroptosis and inhibit myocardial cell death. Additionally, *in vitro* experiments revealed that Calycosin lowered intracellular ROS levels, further confirming its antioxidant and anti-ferroptosis effects.

Nrf2, a vital antioxidant transcription factor, plays a crucial regulatory role in the process of ferroptosis (Morgenstern et al., 2024). Under physiological conditions, Nrf2 forms a dimer with Keap1, resulting in low activity (Morgenstern et al., 2024). When cells encounter oxidative stress, Nrf2 dissociates from Keap1 and translocates to the nucleus to exert its antioxidant effects (Xiang et al., 2024). Core molecules regulated by Nrf2, such as SLC7A11 and GPX4, are downstream proteins, while key proteins involved in glutathione (GSH) production, including glutathione synthetase (GSS) and glutamate-cysteine ligase (GCL), are also regulated by Nrf2 (Psefteli et al., 2023; Zhang et al., 2024; Zhang et al., 2022). Therefore, Nrf2 exerts antioxidant and anti-ferroptosis effects through multiple pathways. Western blot (WB) results indicate that Nrf2 expression increases following doxorubicin intervention, indicating a physiological response to oxidative stress, although the upregulation is limited. Following Calycosin intervention, Nrf2 expression is significantly elevated, and its downstream regulatory molecules, SLC7A11, GPX4, GSS, and GCL, are also markedly upregulated. This enhances the efficiency of GSH production and GPX4 reduction, effectively inhibiting ferroptosis in myocardial cells. *In vitro* immunofluorescence experiments further confirm this finding and highlight the nuclear translocation of Nrf2. These results elucidate the anti-ferroptosis mechanism of Calycosin.

To further validate the central role of Nrf2 in Calycosin’s anti-ferroptosis effects, Nrf2 silencing experiments were conducted. Following the silencing of Nrf2 using siRNA technology, the protective effects of Calycosin were significantly diminished, as evidenced by reduced myocardial cell viability, increased apoptosis rates, and a marked rise in ROS levels. These results indicate that Nrf2 expression is critical for Calycosin’s protective effects on cells. Similar results from Nrf2 silencing experiments were confirmed in studies by Jiang et al. on cardiac injury (Jiang et al., 2022), further supporting the essential role of Nrf2 in regulating the SLC7A11/GPX4 signaling pathway.

Notably, Nrf2, a vital antioxidant transcription factor, assists cells in resisting various stresses by activating the expression of antioxidant genes. The function of Nrf2 is crucial in the pathological processes of heart failure and other diseases. Consequently, silencing Nrf2 not only affects ferroptosis-related pathways but also has profound effects on various other pathways. For instance, silencing Nrf2 significantly reduces the expression of antioxidant genes, including heme oxygenase-1 (HO-1), NAD(P)H quinone oxidoreductase 1 (NQO1), and glutamate cysteine ligase (GCL) (Zhao et al., 2018; El-Mahrouk et al., 2025; Sethi et al., 2024). These genes play critical roles in scavenging free radicals and mitigating oxidative damage. Inhibiting the expression of these genes diminishes the cell’s ability to resist oxidative stress, potentially leading to cellular dysfunction and death, thereby exacerbating the progression of heart failure. Furthermore, Nrf2 is involved in regulating inflammatory responses. When Nrf2 is silenced, the expression levels of inflammatory factors [such as interleukin-6 (IL-6) and tumor necrosis factor-alpha (TNF-α)] within the cells may increase (Kaussikaa et al., 2024; Sheng et al., 2024). This occurs because Nrf2 inhibits the activation of the NF-κB pathway, thereby reducing the production of inflammatory factors (Islam et al., 2024). Silencing Nrf2 leads to enhanced activation of NF-κB, which triggers chronic inflammation, a significant pathological mechanism in diseases such as heart failure. Nrf2 is also closely associated with apoptosis pathways. Silencing Nrf2 may promote apoptosis by enhancing the expression of pro-apoptotic proteins, such as Bax, and reducing the levels of anti-apoptotic proteins, such as Bcl-2 (Sheng et al., 2024). This effect could result in the loss of cardiomyocytes in heart failure, exacerbating the condition. In summary, impaired Nrf2 function may result in a diminished ability of cells to resist various stressors, thereby exacerbating the progression of heart failure and other related diseases. Therefore, future research should investigate the role of Calycosin in other non-ferroptosis-related pathways to further elucidate its potential therapeutic mechanisms.

In the realm of heart failure treatment, Calycosin, a natural compound, has demonstrated significant efficacy, particularly in inhibiting ferroptosis. Compared to other similar compounds and drugs, Calycosin offers distinct advantages and preclinical therapeutic potential. Its mechanism of action shares similarities with several other natural compounds, including resveratrol, curcumin, and ginsenosides. Resveratrol is recognized for its antioxidant and anti-inflammatory effects, having been shown to improve cardiac function and delay the progression of heart failure (Wei et al., 2024; Zhu et al., 2024). In contrast, Calycosin possesses a distinct advantage due to its unique mechanism for inhibiting ferroptosis, enabling direct intervention in the death process of cardiomyocytes (Liu H. et al., 2023; Lu et al., 2021). Curcumin also exhibits significant anti-inflammatory and antioxidant properties, with its application in heart failure primarily focusing on alleviating myocardial injury and improving cardiac function (Abolfazli et al., 2024). However, the low bioavailability of curcumin limits its clinical effectiveness (Pan et al., 2013). In comparison, Calycosin, characterized by its favorable bioavailability and relatively minimal side effects, may present a more effective option for the treatment of heart failure.

Traditional heart failure therapies, including ACE inhibitors, β-blockers, and diuretics, primarily control symptoms by reducing cardiac workload and improving cardiac pumping function; however, they are often accompanied by adverse reactions, such as hypotension and impaired kidney function (Pascual-Figal and Bayes-Genis, 2024). As a supplementary therapy, Calycosin has the potential to enhance patient tolerance and quality of life without causing significant side effects. Further research indicates that Calycosin can enhance the metabolic function of the heart and improve the survival environment of cardiomyocytes, differing from the symptom control primarily targeted by existing medications (Liu H. et al., 2023; Lu et al., 2021). The multiple mechanisms of action of Calycosin suggest broader potential applications in the treatment of heart failure, particularly for patients requiring long-term management of chronic diseases.

In the future, Calycosin could be combined with traditional heart failure therapies to develop a comprehensive treatment strategy. For instance, using Calycosin in conjunction with β-blockers may enhance cardiac protective effects while reducing drug dosages, thereby lowering the risk of potential side effects. Additionally, combining Calycosin with other natural compounds, such as resveratrol or curcumin, could further improve therapeutic effects, creating synergistic benefits and enhancing overall patient health.

However, this study has several limitations. First, a specific rat model was utilized to assess the efficacy of Calycosin. The etiology of heart failure is complex and multifaceted, involving various pathophysiological mechanisms. The selected model is primarily based on doxorubicin-induced myocardial injury, which, while representative in heart failure research, may not fully capture the complexity of human heart failure. Therefore, future studies should consider adopting alternative animal models, such as those involving hypertension or coronary artery disease, to verify the effects of Calycosin across a broader spectrum of heart failure types. Additionally, preclinical studies should explore various animal models to enhance the external validity of the findings.

Second, this study selects rats and H9c2 cardiomyocytes as research models. This choice offers several advantages, including the complete sequencing of the rat genome, which provides a relatively uniform genetic background, and the similarity in physiological characteristics and responses to various drug treatments between rats and humans, thereby offering a reliable foundation for research. Moreover, H9c2 cells effectively simulate the physiological properties of cardiac cells *in vitro*, making them highly suitable for cardiovascular research. However, these two models exhibit notable differences from humans regarding physiological characteristics and responses. Consequently, future research should focus more on these differences and consider adopting other animal species or human-derived cardiomyocyte models to conduct broader and more human-relevant studies.

Although Calycosin demonstrates strong efficacy in inhibiting ferroptosis, its *in vivo* mechanisms may involve multiple targets. Potential off-target effects of Calycosin could result in unanticipated biological outcomes, necessitating further mechanistic studies to elucidate its actions. For instance, Calycosin may influence other signaling pathways, including inflammatory responses and apoptosis, which could impact its specific role in treating heart failure. Therefore, prior to clinical application, systematic biomarker screening and target validation must confirm the specificity of Calycosin.

Finally, our findings suggest that Calycosin holds significant potential for clinical application in treating doxorubicin-induced heart failure. However, clinical translation necessitates further validation. Future research should focus on several aspects: evaluating the drug’s absorption, distribution, metabolism, and excretion characteristics to determine optimal administration routes and dosages; optimizing its efficacy through chemical modifications to enhance its effectiveness and safety in clinical applications; developing new drug carriers, such as hydrogel nanoparticles (Hanafy, 2021), can enhance their affinity for cardiac cells, thereby improving the targeting of the drugs; and conducting clinical trials to further validate its efficacy and mechanisms in humans, with a focus on ensuring safety as the top priority.

## 5 Conclusion

In summary, this study is the first to demonstrate that Calycosin can improve cardiac dysfunction, myocardial injury, and ferroptosis in a rat model of doxorubicin-induced heart failure. The underlying mechanism appears to involve the modulation of ferroptosis through the Nrf2/SLC7A11/GPX4 pathway. These findings suggest that Calycosin holds promise as a therapeutic agent against ferroptosis and heart failure, warranting further investigation. Additionally, this study utilized a specific rat model based on doxorubicin-induced myocardial injury, which may not fully capture the complexity of human heart failure. Future research should explore a broader array of animal models and focus on the *in vivo* mechanisms of Calycosin, including potential off-target effects and interactions with other signaling pathways. Moreover, systematic biomarker screening and clinical trials are essential to validate its efficacy, safety, and optimal administration methods in treating heart failure.

## Data Availability

The original contributions presented in the study are included in the article/supplementary material, further inquiries can be directed to the corresponding authors.

## Abbreviations

HF, Heart failure; NT-Pro BNP, N-Terminal Pro-Brain Natriuretic Peptide; LVEF, Left ventricular ejection fraction; TEM, Transmission electron microscope; Nrf2, Nuclear factor erythroid 2-related factor 2; GPX4, Glutathione peroxidase 4; SLC7A11, Solute carrier family 7 member 11; GSS, Glutathione synthetase; GCL, Glutamate-cysteine ligase; GSH, Glutathione; Keap1, Kelch-like ECH-associated protein 1; AREs, Antioxidant response elements; DMSO, Dimethyl sulfoxide; SBE-β-CD, Sulfobutylether-β-Cyclodextrin; GSSH, Glutathione disulfide; MDA, Malondialdehyde; ROS, Reactive oxygen species.
